# A Rare, Unreported Cognitive Side Effect of Topiramate: Do We Know It All Yet?

**DOI:** 10.7759/cureus.11342

**Published:** 2020-11-05

**Authors:** Nurose Karim, Ajaz A Sheikh

**Affiliations:** 1 Neurology, University of Texas Southwestern Medical Center, Dallas, USA; 2 Neurology, University of Toledo Medical Center, Toledo, USA

**Keywords:** topiramate, naranjo algorithm, serotonin, cognitive side effects, substance induced dissociative amnesia

## Abstract

This report describes a unique, dose dependent side effect of a commonly used drug, topiramate. Although cognitive side effects of this drug have previously been reported in literature, we present a case of drug-induced amnesia, with support from Naranjo Nomogram, as a hitherto unreported side effect of topiramate. Here, we highlight the importance of being cognizant of such rare cognitive side effects, with the aim of improving patient outcome by timely recognition, and discontinuation of the offending drug, as the side effect was fortunately found to be reversible.

## Introduction

2,3:4,5-di-O-isopropylidene-β-d-fructopyranose sulfamate, commonly known as topiramate, is a well-known anti-epileptic drug (AED) with a broad mechanism of action. It is approved for both partial and generalized seizures [1]. It is also frequently used off-label for migraine prophylaxis. We present a case of a woman who experienced hitherto unreported, and fortunately reversible, cognitive side effect from topiramate use.

## Case presentation

A 25-year-old right-handed woman with no significant medical history other than migraine headaches, was evaluated by her neurologist in September 2018. She was started on topiramate ER 100 mg daily for migraine prophylaxis. In October, she started experiencing episodes of amnesia that lasted from 30 minutes to several hours. The period she was amnestic to, extended from six months to six years, in a retrograde fashion. She was completely aware of the events, and was very distressed by them. These episodes were not related to headaches and were otherwise apparently un-triggered. Frequency of these episodes started increasing progressively with increase in topiramate dose. She had a total of 20 amnestic episodes, before they ceased to happen. With a concern for seizures, she was admitted for video-electroencephalographic monitoring for three days, which was normal. MRI brain with and without contrast was also normal. Transient ischemic attack was low in the differentials since she did not have any vascular risk factors and some of these episodes lasted more than 24 hours. A tentative diagnosis of dissociative amnesia was made, and she was evaluated for symptoms of depression and other psychopathology, and none were appreciated. Since she was only taking topiramate, it was suspected to be likely related to her symptoms. She was gradually weaned off topiramate, until it was discontinued in January 2019. She had her final amnestic spell one day after the last dose of topiramate, which lasted about 45 minutes with amnesia extending back for 3.5 years. She has subsequently been completely free of the amnestic spells and is periodically following-up with us.

## Discussion

Topiramate is a potent AED that was approved in the Federal Republic of Germany in July 1998, and ever since it has been used in various neurological and non-neurological conditions due to its broad mechanism of action [1]. It acts on voltage gated sodium and calcium channels, inhibits excitatory glutamate and enhances inhibitory effects of gamma-aminobutyric acid (GABA). It has been used for partial and generalized seizures either by itself, or as an add-on with other AEDs. A pooled analysis of data by Reife et al. in August 2005 showed that topiramate, when used as an add-on therapy, decreased the seizure frequency by greater than 50% in 43% of the cases, with 21% showing reduction in seizure frequency by up to 75%. About 5% of patients who were previously labelled drug-resistant, became seizure-free [2]. This data provides evidence supporting the efficacy of this drug.

Other uses of topiramate include migraine headaches, bipolar disorder, post-traumatic stress disorder, chronic alcoholism and treatment of obesity. The most common side effects (SE), which accompany the use of topiramate, are fatigue, paresthesia, decreased appetite, nausea, diarrhea, weight loss and taste perversion. Neurocognitive side effects include mental slowing, word-finding difficulty, agitation and irritability [3-5]. Despite being an attractive drug, it has negative (loss of function) effects on cognition such as, slowing of the thought process, difficulty in mental tasks such as calculation and decision making, blunt affect and slow reaction time. Aldenkamp et al. showed that the major cognitive impairment was difficulty focusing and trouble with verbal tests [6]. Kockelmann et al. demonstrated that significant improvement occurred in cognition after discontinuing topiramate treatment [7]. Reviewing the study by Tatum et al. approximately 41% patients on topiramate experienced central nervous system (CNS)-related side effects at least once, during their course of treatment [8].

In a study comparing the efficacy of three different AEDs, topiramate, gabapentin and lamotrigine for the treatment of seizures, greater impact on attention, concentration and speech difficulty was seen in young adult patients [9]. Incidence of cognitive side effects was 33.3% in the first month, and 20.8% in the second month. These specifically included impaired attention and concentration, difficulty with calculations, slowing of the thought process and word-finding difficulty (deterioration in phonematic as well as semantic word fluency). Aldenkamp et al. compared cognitive side effects of topiramate and Valproic acid, when used in adjunction to Carbamazepine in patients with refractory partial seizures [6]. Short-term memory, evaluated by Recognition of Word test, showed decreased score with both topiramate and Valproic acid, although, effect of topiramate was greater. Interestingly, learning and memory were affected very little by topiramate. The only memory issue seen was delayed recall.

The etiology for CNS-related side effects has been argued and debated in various studies. There has been no mutual consensus so far, and the likely risk factors could be anything from idiosyncratic side effects to total topiramate dose, to high initial dose and/or rapid dose titration [8,10]. There was a case series on two patients, reported by Stella et al. where they reported reversible psychotic side effects with topiramate [10]. Both patients had no known psychiatric history. Topiramate was added to as an add-on therapy to Carbamazepine in the first patient, and Valproic acid in the second, for seizure control. Symptoms started suddenly on higher dose (350 mg once a day in the first patient and 150 mg twice a day in the second patient). The symptoms resolved completely after stopping topiramate.

As per Diagnostic and Statistical Manual, version 5 (DSM-5), substance-induced dissociative amnesia is defined as an inability to recall important autobiographical information, usually of a traumatic or stressful nature, that is inconsistent with ordinary forgetting and is secondary to some external factor (drug influence) [11-12]. Dissociative disorder is an independent co-occurring mental disorder which can occur in someone with primary mental disorder but to label it as substance-induced amnesia, the primary cause should be substance use, abuse, intoxication, or withdrawal than of underlying mental illness. Cocaine, methamphetamine, hallucinogens, opioids and sedatives are well known to cause substance-induced amnesia [13].

Our case describes topiramate causing dissociative amnesia which has never been reported previously. There are several pointers that implicate topiramate as the culprit. The patient had no previous psychiatric history, and no subsequent psychiatric symptoms. Her symptoms started shortly after initiation of topiramate and progressively worsened with dose escalation. And, finally the symptoms completely resolved after discontinuation of topiramate. Naranjo algorithm is a questionnaire designed by Naranjo et al. for determining the likelihood of an adverse reaction to be due to a drug rather than other factors [13]. Our patient scored 6 points on the algorithm (‘probable’ on the probability scale), meaning, her symptoms were probably an adverse reaction from topiramate.

The exact mechanism of topiramate causing dissociative symptoms is unknown. Sarwar and McGinnis proposed a potential link between elevated plasma levels of serotonin and dissociative amnesia [14]. Interestingly, topiramate has been shown to increase plasma levels of serotonin [2]. In 2008, a study was done on 12 children between the ages of 2-12 year, who were on topiramate for seizures [15]. After three months of treatment, their plasma serotonin levels were measured and were found to be significantly elevated. This explains the utility of topiramate in various eating disorders such as bulimia nervosa and treatment of alcohol dependence. Hallucinogens such as lysergic acid diethylamide, mescaline, and dimethyltryptamine, also produce dissociative symptoms due to their action on serotonin receptors (5-HT2 receptor agonism). This could be one reasonable explanation for dissociative symptoms in our case. Having said that, more research is needed to support this hypothesis.

## Conclusions

Despite topiramate’s wide availability and usage, its use can sometimes be limited due to adverse effects. It is not surprising that, based on its multiple mechanisms of action, it can cause unusual cognitive side effects, one rare form of which is reversible, episodic dissociative symptoms, possibly secondary to increased plasma serotonin levels. Physicians must be mindful of such rare cognitive side effects that may necessitate discontinuation of drug, with close follow-up.
